# Lymphotoxin β receptor activation promotes bladder cancer in a nuclear factor-κB-dependent manner

**DOI:** 10.3892/mmr.2014.2826

**Published:** 2014-10-30

**Authors:** MO SHEN, XIUZHI DUAN, PING ZHOU, WU ZHOU, XIULING WU, SIQI XU, YUHUA CHEN, ZHIHUA TAO

**Affiliations:** 1 Department of Laboratory Medicine, The First Affiliated Hospital of Wenzhou Medical University, Zhejiang 325000, P.R. China; 2 Department of Laboratory Medicine, The Second Affiliated Hospital, Zhejiang University College of Medicine, Hangzhou 310009, P.R. China; 3 Wenzhou WuMa Community Health Service Center, Wenzhou 325000, P.R. China; 4 Department of Pathology, The First Affiliated Hospital of Wenzhou Medical University, Zhejiang 325000, P.R. China

**Keywords:** bladder cancer, lymphotoxin β receptor, NF-κB pathway

## Abstract

Bladder cancer (BCa) is the most common tumor of the urinary system. Chronic inflammation in the papillary urothelial neoplasm of low malignant potential (PUNLMP)may contribute to carcinogenesis, including that of BCa, via poorly understood mechanisms. In this study, we show that the lymphotoxin β receptor (LTβR) is upregulated in BCa via activation of the canonical and non-canonical nuclear factor-κB (NF-κB) pathways. The mRNA expression of LTβR in 81 BCa, 10 chronic cystitis and 23 healthy bladder mucosa tissues was investigated by reverse transcription-fluorescent quantitative polymerase chain reaction (RT-FQ-PCR), and protein expression was studied in 73 BCa, 30 cystitis and 15 healthy paraffin-embedded tissue sections by immunohistochemistry. Both LTβR mRNA and protein were upregulated in BCa and cystitis compared to the healthy group (P<0.05). The mRNA level of the downstream NF-κB canonical pathway p65 gene and of the non-canonical pathway RelB gene were higher in the BCa and cystitis groups compared to the healthy one. The level of phosphorylated p65 (p-p65) protein of the canonical NF-κB pathway and that of p52, a protein of the non-canonical NF-κB pathway, were also higher in the BCa and cystitis group compared to the healthy group. The levels of these proteins significantly correlated to the pathological grade, clinical stage and lymph node metastasis of BCa patients (P<0.05). In addition, there was a positive correlation between LTβR and NF-κB pathway proteins. Thus, LTβR signaling may be involved in promoting BCa through the NF-κB pathway, and which may represent the molecular link between inflammation and BCa.

## Introduction

Bladder carcinoma is the most common malignant neoplasia of the urinary tract (1), and is characterized by wide prognostic variability. Carcinomas of transitional cells account for >90% of bladder tumors (2). It is well known that smoking is the most important risk factor for bladder cancer (BCa). Workers in the dye, rubber, or leather industries and individuals who live in communities with high levels of arsenic in the drinking water are also high-risk groups (1). However to date, the mechanisms associated with the initiation and progression of these tumors are not well understood. Chronic inflammation has long been suggested to constitute a risk factor for a variety of epithelial cancers, including BCa (3). The relationship between inflammation and cancer is an important research focus since the end of the nineteenth century (4). More than 15% of malignancies worldwide are attributed to infections, totaling to a 1.2 million cases per year (5,6). In certain types of cancer, inflammatory conditions are present prior to the occurrence of malignant change. Conversely, in other types of cancer, an oncogenic change creates an inflammatory microenvironment that promotes the development of tumors (7). Although numerous studies have suggested that inflammation may contribute to bladder tumor growth, the underlying mechanisms have not yet been fully elucidated (8). The molecular pathways of cancer-related inflammation are now being unraveled, resulting in the identification of new target molecules that may allow improved diagnosis and treatment (7).

The lymphotoxin β receptor (LTβR), a member of the tumor necrosis factor (TNF) receptor superfamily, is expressed on the surface of most cell types, including epithelial cells. A recent study provided strong evidence for an involvement of LTβR signaling in inflammatory processes and carcinogenesis, by demonstrating that the LTβR signaling pathway can initiate inflammation-induced carcinogenesis and affect primary tumorigenesis, as well as control the reemergence of carcinoma in various cancer models through distinct mechanisms (9). LTβR was shown to be expressed in several solid tumors (10). The LTβR signaling pathway plays an important role in inflammation-induced cancer processes, including the transformation from hepatitis to liver cancer (11), and prostate cancer occurrence and metastasis (12,13). LTβR is an upstream activator of nuclear factor-κB (NF-κB)-mediated transcription (14–16). The activation of LTβR by LTα1β2 (a cell-bound heterotrimeric complex of LTα and LTβ) and LIGHT promotes tumor growth in an NF-κB-dependent manner (17). Signals from LTβR can induce chemokine or cytokine expression, cell proliferation and survival through non-canonical/canonical NF-κB pathways (9). NF-κB is a family of ubiquitously expressed transcription factors that is crucial in inflammatory signaling (18), and the key pathway connecting inflammation and cancer, by inducing cell proliferation, tumor invasion and apoptosis (3). In addition, NF-κB functions as a tumor promoter in inflammation-associated cancer (19). p65 is one of the canonical NF-κB pathway proteins, while RelB and p52 are components of the non-canonical NF-κB pathway.

In the present study, we investigated the expression of LTβR pathway-related genes and proteins in BCa, chronic cystitis and healthy bladder mucosa tissues. We aimed to identify a potential causal relationship between cystitis, LTβR signaling and BCa development.

## Materials and methods

### Patient cohort

Fresh tissue specimens from 114 outpatients and inpatients were collected and stored in liquid nitrogen for analysis. Subjects were grouped as follows: BCa transitional cell carcinoma (TCC) patient group (n=81), chronic cystitis patient group (n=10), and healthy bladder mucosa group (n=23). TCC was classified as Ta + T1 (n=46) or ≥T2 (n=35), and histologically graded as papillary urothelial neoplasm of low malignant potential (PUNLMP) + G1 (n=26), G2 (n=28) or G3 (n=27). Formalin-fixed, paraffin-embedded tissue sections were collected from 118 patients, including 73 BCa patients [classified as Ta + T1 (n=37) or ≥T2 (n=36), and graded as PUNLMP + G1 (n=32), G2 (n=20) or G3 (n=21)], 30 cystitis patients and 15 healthy individuals.

BCa and cystitic tissues were obtained from patients who underwent transurethral bladder tumor resection or partial/radical cystectomy. The healthy bladder mucosa was derived from traumatic bladder ruptures of patients confirmed to have no cancer and cystitis and was used as a control, since the majority of adjacent tissues to BCa were found to be cystitic or bladder inverted papilloma with cystitis at postoperative pathological examinations, and were thus not appropriate. All tumors were confirmed by histopathological evaluation, classified according to the World Health Organization system for the classification of tumors (1973/2004) (20,21), and the tumor-nodes-metastasis (TNM) classification system of the Union International Against Cancer (22). Approval was obtained from the Medical Ethics Committee of the First Affiliated Hospital of Wenzhou Medical University, and written informed consent was obtained from all patients.

### Reverse transcription-fluorescent quantitative polymerase chain reaction (RT-FQ-PCR)

Total RNA was extracted from the fresh tissues using the Invitrogen^®^ TRIzol Reagent (Thermo Fisher Scientific, Waltham, MA, USA), and cDNA was synthesized using the RevertAid™ First Strand cDNA Synthesis kit (Thermo Fisher Scientific). The cDNA was stored at −20°C until further use.

The primers for the FQ-RT-PCR amplification of LTβR, p65 (RelA) and RelB are listed in Table I. PCR amplification was performed on the Applied Biosystems^®^ ABI Prism^®^ 7000 system (Thermo Fisher Scientific), and the reaction system contained 200 nmol/l of the forward primer, 200 nmol/l of the reverse primer, 2 μl cDNA and 10 μl Invitrogen Platinum^®^ SYBR^®^-Green qPCR SuperMix-UDG (Thermo Fisher Scientific) in a final volume of 20 μl. Following an activation step at 95°C for 2 min, 40 cycles of amplification were performed at 95°C for 15 sec, and 60°C for 30 sec. The glyceraldehyde 3-phosphate dehydrogenase gene (GAPDH) was selected as an internal reference for the quantification of the expression of target genes. The relative mRNA level of target genes was calculated as follows:

$$\frac{Target\;gene\;mRNA}{GAPDH\;mRNA}=2^{-(Target\;gene\;mRNA.Ct-GAPDH\;mRNA.Ct)}$$

### Immunohistochemistry

The tissue sections were incubated with the 48-kDa rabbit polyclonal antibody against LTβR (Ab70063; Abcam, Cambridge, MA, USA) used at a 1:120 dilution, the 65-kDa rabbit polyclonal antibody against phosphorylated (p)-NF-κB p65 (Ser536) (sc-101752; Santa Cruz Biotechnology, Inc., Santa Cruz, CA, USA) used at a 1:80 dilution, and the 54-kDa rabbit polyclonal antibody against NF-κB p100/p52 (Ab31409; Abcam) used at a 1:100 dilution. A total of 118 representative 3-μm sections of formalin-fixed, paraffin-embedded tumor tissues were obtained. Briefly, sections were dewaxed with xylene and hydrated in a series of graded alcohol solutions. Endogenous peroxidase activity was blocked with addition of 0.3% hydrogen peroxidase for 20 min. After blocking with 10% goat serum for 30 min, the sections were incubated with LTβR antibody, at a 1:120 dilution; p-p65 antibody, at a 1:80 dilution; and 100/p52 antibody, at a 1:100 dilution. Sections were then incubated overnight at 4°C. Next, the sections were rinsed in water, counterstained, dehydrated and mounted in neutral balsalm. Liver cancer tissue was used as a positive control for LTβR expression and breast cancer tissue for p-p65 and p52 expression, while phosphate-buffered saline (PBS) instead of the primary antibody was used as the negative control.

Evaluation of staining intensities was performed under an optical microscope (CX31-LV320; Olympus Corporation, Tokyo, Japan) by different observers, and the results were graded as follows: no staining or staining observed in <10% of cells, score 0; not/barely perceptible staining detected in ≥10% of cells, 1^+^; moderate or strong to complete staining observed in ≥10% of cells, 2^+^–3^+^. Scores of 0 and 1^+^ were considered negative, whereas 2^+^ and 3^+^ were considered positive. The Image-Pro Plus (IPP) 6.0 software (Media Cybernetics, Inc., Rockville, MD, USA) was used to quantify the intensity of the stained sections, and the mean density (MD) value was averaged from five fields of view. All images analyzed with IPP 6.0 were counter-checked by a pathologist. MD was calculated as follows (IOD, integrated optical density):

$$\begin{array}{l} Mean\;density\;(MD)=\frac{IOD}{area}=\frac{\int_{s}density(x,y)ds}{area}\\ x=\text{area},\;y=\text{density} \end{array}$$

### Statistical analysis

Statistical analysis was performed using the Statistical Package for Social Sciences (SPSS), version 17.0 (SPSS, Inc., Chicago, IL, USA). The expression of the genes LTβR, p65 and RelB was expressed as median value and range. The statistical significance of differences in the relative mRNA levels of these genes among the three groups (healthy bladder mucosa, chronic cystitis and BCa) was evaluated with the nonparametric Kruskal-Wallis H test, due to skewed distributions of corresponding values. A Mann-Whitney U test was used to analyze the differences in mRNA levels between two groups. The nonparametric Kruskal-Wallis H test was also used to examine the mRNA level differences among different histological grade BCa patients, while the Mann-Whitney U test was used to analyze the differences among groups classified based on the remaining clinical indicators.

Differences in the LTβR, p65 and p52 protein levels were evaluated with a one-way analysis of variance (ANOVA) using the MD values, and a Student-Newman-Keuls test was used to analyze the expression between two groups. One-way ANOVA was also used to examine the protein differences among various histological grade BCa patients, and an independent-samples t-test was used to statistically analyze protein expression differences among groups classified based on the remaining clinical indicators. Correlation analysis of data showing a normal and a skewed distribution was performed with the Pearson correlation and the Spearman’s rank correlation, respectively. P-values <0.05 were considered to indicate statistical significance.

## Results

### Expression levels of LTβR, p65 and RelB mRNA in BCa tissues

The expression of the *LTβR* gene in the BCa and the chronic cystitis groups was higher than that observed in the healthy bladder mucosa group, as revealed by Mann-Whitney U tests (both, P<0.05). Moreover, the LTβR level was significantly different between the histological grade, the clinical stage, and lymph node metastasis profile groups of BCa patients (all, P<0.05), but not significantly different (both, P>0.05) between age and gender groups (Tables II and III).

The p65 and RelB mRNA levels were both higher in the BCa and the chronic cystitis group compared to the healthy bladder mucosa group (both, P<0.05). They were significantly different between different histological grade groups of BCa patients (both, P<0.05), different clinical stages (both, P<0.05), and different lymph node metastasis profiles (both, P<0.05). No significant difference was observed in between different age or gender groups (both, P>0.05) (Tables II and III).

### Expression of LTβR, p-p65 and p52 proteins in BCa tissues

**Figure 1:**
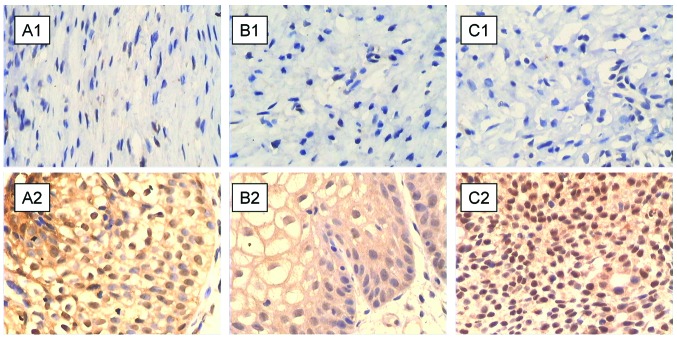
Immunohistochemical staining of (A) lymphotoxin β receptor (LTβR), (B) phosphorylated (p)-p65, and (C) p52 proteins in (A1–C1) healthy bladder mucosal and (A2–C2) chronic cystitis tissues. All three proteins are negatively stained in healthy bladder mucosa tissues and show strong positive staining in chronic cystitis tissues (×400).

**Figure 2:**
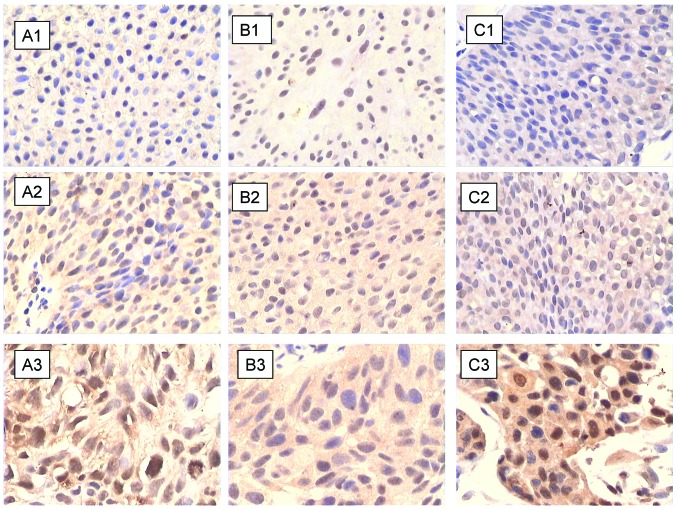
Immunohistochemical staining of (A) lymphotoxin β receptor (LTβR), (B) phosphorylated (p)-p65, and (C) p52 proteins in different bladder cancer (BCa) pathologic grades: (A1–C1) G1, (A2–C2) G2 and (A3–C3) G3. All three proteins show weak positive staining in G1-, positive staining in G2-, and strong positive staining in G3-grade BCa tissues (×400).

Weak to no LTβR protein expression was detected in healthy bladder mucosa, while positive staining was observed in the cytoplasm and nucleus of chronic cystitis and BCa tissues (Figs. 1 and 2). Positive staining of the LTβR protein (69.8%) in the BCa group was higher than that in healthy bladder mucosa one (13.3%), similarly to the cystitis (90.0%) group. The MD of the LTβR protein in the BCa group was significantly higher than that observed in the healthy group, as shown by a Student-Newman-Keuls test (P<0.05), similarly to the comparison between the chronic cystitis and the healthy group (P<0.05) (Table IV). The MD values of LTβR were significantly different between the histological grade, the clinical stage and the lymph node metastasis profile groups of BCa patients (all, P<0.05), while no significant association with age and gender (P>0.05) was observed (Table V and Fig. 2).

The p-p65 and p52 proteins appeared not or weakly expressed in healthy bladder mucosa, while positive staining was clearly observed in the cytoplasm and nucleus of chronic cystitis and BCa tissues (Figs. 1 and 2). Positive staining of the p-p65 and p52 proteins in the BCa group (56.2 and 63.0%, respectively) was higher than that in the corresponding healthy groups (6.7 and 6.7%, respectively), a result similar to the cystitis (83.3 and 93.3% for p-p65 and p52, respectively) and healthy group comparison. The MD values of p-p65 and p52 were higher compared to the healthy group in both the BCa and the chronic cystitis group, as shown by the Student-Newman-Keuls tests (both, P<0.05) (Table IV). In addition, the MD values of the two proteins were significantly different between the histological grade, the clinical stage and the lymph node metastasis profile groups of BCa patients (all, P<0.05), while no statistically significant difference was observed between the different age or gender groups (both, P>0.05) (Table V and Fig. 2).

### Correlation between LTβR and NF-κB

**Figure 3:**
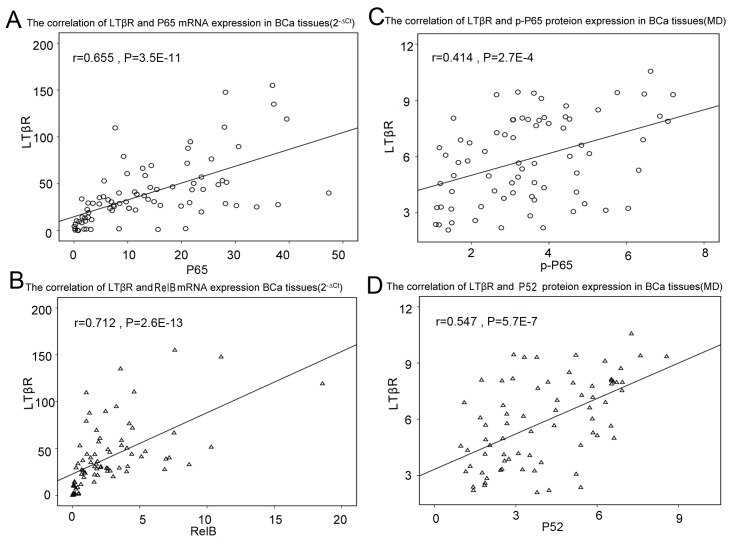
Correlations between lymphotoxin β receptor (LTβR) and nuclear factor-κB (NF-κB) pathway in bladder cancer (BCa) tissues. Correlation between LTβR and (A) *p65* mRNA (r=0.655, P<0.001), (B) *RelB* mRNA (r=0.712, P<0.001), (C) phosphorylated (p)-p65 protein (r=0.414, P<0.001), and (D) p52 protein (r=0.547, P<0.001). MD, mean density; Ct, cycle threshold.

Since LTβR and NF-κB appear simultaneously upregulated in BCa, and previous studies (9,14,17) have indicated that LTβR acts as an upstream activator of NF-κB in numerous carcinoma cells, we explored the correlation between the two proteins in BCa tissues, using Pearson and Spearman’s rank correlation tests. We found a positive correlation between the mRNA and protein levels (MD values) of LTβR and the classical NF-κB pathway p65 subunit in BCa (r=0.655, P<0.001; and r=0.414, P<0.001, respectively). LTβR also positively correlated to the non-canonical NF-κB pathway RelB protein at the mRNA level (r=0.712, P<0.001) and the p52 protein (r=0.547, P<0.001) at the protein level in BCa (Fig. 3). In addition, our early research demonstrated that the protein expression of LTβR is positively correlated to that of p-p65 and p52 in chronic cystitis tissues. Thus, LTβR appears associated with NF-κB in BCa, which supports its potential role as an upstream activator of the NF-κB pathway to promote BCa. The correlation between LTβR and NF-κB in BCa was similar to that observed in chronic cystitis, which suggests that BCa may be related to inflammation. This result thus provides useful insights for the understanding of the mechanism by which inflammation can lead to BCa.

## Discussion

In the last decade, studies have corroborated the tight link between chronic inflammation and carcinogenesis. Consequently, the inflammatory microenvironment was added as the seventh hallmark of cancer (23,24). In parallel, the role of the LTβR signaling pathway in cancer has been recognized (10–13). However, little is known regarding the expression of LTβR in human BCa tissue and the role of inflammation in the occurrence and progression of BCa. Here, we focused on the LTβR protein and its involvement in BCa.

In this study, we found that the mRNA and protein expression of LTβR were significantly higher in the BCa group compared to the healthy bladder mucosa group, and that this increase is associated with the pathological grade, clinical stage and lymph node metastasis profile of BCa tissues (Tables II and III). Higher expression of LTβR compared to matching healthy tissues was also reproduced in the BCa T24 cell line (data not shown).

NF-κB is a family of ubiquitously expressed transcription factors that are considered to trigger both the onset and the progress of inflammation. p65 is one of the canonical NF-κB pathway proteins, with its serine 536 being the key phosphorylation site for the activation of this pathway (25,26). LTβR is an upstream activator of NF-κB that can activate the canonical NF-κB pathway in a number of carcinomas (11,27). Therefore, we detected the expression of the canonical NF-κB pathway protein p65, and showed that the p65 mRNA and phosphorylated protein (p-p65) level were both increased in BCa tissues. Moreover, a positive correlation was detected between the two proteins in the BCa group, at both the mRNA and activated protein levels. These results suggest that LTβR may activate the classical NF-κB pathway in BCa. In addition, a number of studies have reported that LTβR is involved in tumor angiogenesis, most likely through the non-canonical NF-κB pathway (28). The RelB and p52 proteins are involved in the non-canonical NF-κB pathway. In this view, we examined the *RelB* mRNA and p52 protein levels in BCa tissues, and found that *RelB* mRNA and p52 protein levels are also more highly expressed in BCa tissues compared to the healthy ones. *RelB* mRNA and p52 protein expression were also associated with the BCa tumor grade, clinical stage and lymph node metastasis profile. Furthermore, Spearman’s rank correlation tests revealed a positive correlation between *RelB* and *LTβR* mRNA levels and between the p52 and LTβR protein levels in the BCa groups. Therefore, the LTβR and the non-canonical NF-κB pathway also appear to associate in the context of BCa development. Moreover, the correlation between LTβR and the non-canonical NF-κB pathway proteins was higher than that between LTβR and the canonical NF-κB pathway protein p65, which indicates that LTβR may promote BCa through the non-canonical NF-κB pathway.

The present study confirmed the high expression of LTβR in BCa and the association of this protein with other signaling molecules involved in the pathogenesis and progress of BCa, which suggests that the LTβR signaling pathway may be involved in BCa development. Our results further show that LTβR promotes BCa through the NF-κB pathway, and most likely, predominantly through the non-canonical NF-κB pathway. It is unknown whether LTβR, as an inflammatory factor receptor, is the key molecule that links cystitis to BCa, or only acts upstream of NF-κB. We showed that the mRNA and protein of LTβR are both increased in the BCa tissues, similar to the cystitic ones and in contrast to the healthy bladder mucosa tissues, where LTβR was lowly expressed. However, there is no direct evidence of the association between cystitis and BCa, and the LTβR may be critical for further investigation. Additional studies with increased sample size, and research on BCa cell lines are needed to assess the clinical relevance of the LTβR pathway.

## Figures and Tables

**Table I:** Primer sequences used for reverse transcription-fluorescence quantitative polymerase chain reaction (RT-FQ-PCR).

Gene	Forward sequence (5′-3′)	Reverse sequence (5′-3′)
*LTβR*	GCACAAGCAAACGGAAGACC	GACCTTGGTTCTCACACCTGGT
p65 *(RelA)*	GTGGGGACTACGACCTGAAT	GGGGCACGATTGTCAAAGAT
*RelB*	CGTCTATGACAAGAAATCCACAAAC	GACAATCTCCAGGTCCTCGTA
*GAPDH*	GTCAACGGATTTGGTCGTATTG	CTGGAAGATGGTGATGGGATT

LTβR, lymphotoxin β receptor; GAPDH, glyceraldehyde 3-phosphate dehydrogenase.

**Table II:** *LTβR*, *p65 (RelA)* and *RelB* mRNA expression in the healthy bladder mucosa, chronic cystitis and BCa groups.

Group (N)	*LTβR*/*GAPDH* mRNA ×10^3^ median (P_25_-P_75_)	*p65*/*GAPDH* mRNA ×10^3^ median (P_25_-P_75_)	*RelB*/*GAPDH* mRNA ×10^3^ median (P_25_-P_75_)
Healthy bladder mucosa (23)	1.2 (0.3–7.0)	1.0 (0.8–1.8)	0.2 (0.04–0.2)
Chronic cystitis (10)	6.8 (1.3–22.7)^a^	2.8 (1.9–7.1)^a^	0.4 (0.1–0.9)^a^
BCa (81)	29.8 (16.8–50.9)^a^,^b^	10.2 (3.2–21.8)^a^,^b^	1.8 (0.5–3.6 )^a^,^b^

a P<0.05 compared to the healthy bladder mucosa group;

b P<0.05 compared to the healthy bladder mucosa and chronic cystitis group.

LTβR, lymphotoxin β receptor; BCa, bladder cancer; GAPDH, glyceraldehyde 3-phosphate dehydrogenase; P_25_–P_75_, percentile 25–75%.

**Table III:** *LTβR*, *p65* (*RelA*) and *RelB* mRNA expression in the different BCa groups classified by clinical indicator.

Clinical indicator (N)	*LTβR*/*GAPDH* mRNA ×10^3^ median (P_25_–P_75_)	*p65*/*GAPDH* mRNA ×10^3^ median (P_25_–P_75_)	*RelB*/*GAPDH* mRNA ×10^3^ median (P_25_–P_75_)
Age (years)			
≥65 (45)	26.8 (10.1–55.2)	10.2 (2.9–23.8)	1.2 (0.3–3.6)
<65 (36)	33.5 (23.7–50.1)^a^	10.9 (3.8–21.4)^a^	1.8 (0.9–3.6)^a^
Gender			
Male (62)	30.3 (17.9–49.6)	10.8 (3.5–22.5)	1.8 (0.5–3.7)
Female (19)	28.4 (10,0–57.1)^a^	9.6 (2.5–21.2)^a^	1.6 (0.4–2.6)^a^
Histological grade			
PUNLMP + G1 (26)	18.2 (2.1–31.3)	4.9 (1.3–12.0)	0.5 (0.1–1.6)
G2 (28)	28.6 (19.7–42.2)	8.9 (3.3–22.1)	1.8 (0.7–3.6)
G3 (27)	49.0 (33.7–76.4)^b^	21.1 (10.2–28.2)^b^	3.3 (1.8–6.9)^b^
Clinical stage			
Ta + T1 (46)	25.9 (10.3–31.9)	7.2 (2.2–15.0)	1.1 (0.2–2.6)
≥T2 (35)	43.6 (30.6–66.5)^b^	16.1 (9.8–24.0)^b^	2.6 (1.0–4.7)^b^
Lymph node metastasis			
Negative (49)	27.2 (9.9–41.8)	7.4 (2.4–20.7)	1.6 (0.2–2.6)
Positive (32)	40.7 (26.7–56.2)^b^	14.0 (8.2–23.3)^b^	2.3 (0.9–5.2)^b^

a P>0.05;

b P<0.05.

LTβR, lymphotoxin β receptor; BCa, bladder cancer; GAPDH, glyceraldehyde 3-phosphate dehydrogenase; PUNLMP, papillary urothelial neoplasm of low malignant potential; P_25_–P_75_, percentile 25–75%.

**Table IV:** LTβR, p-p65 and p52 protein expression in the healthy bladder mucosa, chronic cystitis and BCa groups.

	LTβR		p-p65		p52	
			
Group (N)	Positive rate (%)	MD ×10 (mean ± SD)	Positive rate (%)	MD ×10 (mean ± SD)	Positive rate (%)	MD ×10 (mean ± SD)
Healthy bladder mucosa (15)	13.3	2.7±0.8	6.7	1.7±0.7	6.7	1.9±0.9
Chronic cystitis (30)	90.0	9.0±2.2^a^	83.3	5.8±1.5^a^	93.3	6.6±1.7^a^
BCa (73)	69.8	5.9±2.3^a^,^b^	56.2	3.5±1.6^a^,^b^	63.0	4.0±2.0^a^,^b^

a P<0.05 compared to the healthy bladder mucosa group;

b P<0.05 compared to the healthy bladder mucosa and chronic cystitis group.

LTβR, lymphotoxin β receptor; p-p65, phosphorylated p65; BCa, bladder cancer; MD, mean density; SD, standard deviation.

**Table V:** LTβR, p-p65 and p52 protein expression in the different BCa groups classified by clinical indicator.

	LTβR		p-p65		p52	
			
Clinical indicator (N)	Positive rate (%)	MD ×10 (mean ± SD)	Positive rate (%)	MD ×10 (mean ± SD)	Positive rate (%)	MD ×10 (mean ± SD)
Age						
≥65 (41)	78.0	6.1±2.3	53.7	3.4±1.6	61.0	3.8±2.0
<65 (32)	59.4	5.6±2.3^a^	59.4	3.6±1.6^a^	65.6	4.3±1.9^a^
Gender						
Male (54)	70.4	6.0±2.3	57.4	3.6±1.6	63.0	4.1±2.0
Female (19)	68.4	5.6±2.2^a^	52.6	3.0±1.8^a^	63.2	3.9±1.9^a^
Histological grade						
PUNLMP + G1 (32)	56.3	4.6±1.7	40.6	2.6±1.3	50.0	3.2±1.7
G2 (20)	75.0	6.5±2.0	60.0	3.6±1.3	60.0	4.3±1.8
G3 (21)	85.7	7.3±2.3^b^	76.2	4.7±1.6^b^	85.7	5.1±2.1^b^
Clinical stage						
Ta + T1 (37)	54.1	4.8±1.9	40.5	2.8±1.4	43.2	3.4±1.7
≥T2 (36)	86.1	6.9±2.2^b^	72.2	4.1±1.6^b^	83.3	4.7±2.0^b^
Lymph node metastasis						
Negative (48)	58.3	5.2±2.1	52.1	3.1±1.4	54.2	3.6±1.9
Positive (25)	92.0	7.2±2.3^b^	64.0	4.2±1.8^b^	80.0	4.8±1.9^b^

a P>0.05;

b P<0.05.

LTβR, lymphotoxin β receptor; p-p65, phosphorylated p65; BCa, bladder cancer; MD, mean density; SD, standard deviation; PUNLMP, papillary urothelial neoplasm of low malignant potential.
